# An Atypical Adverse Local Tissue Reaction After Ceramic-on-Ceramic Primary Total Hip Arthroplasty

**DOI:** 10.1016/j.artd.2022.01.025

**Published:** 2022-02-28

**Authors:** Kamran Movassaghi, Arpan Patel, Ira Miller, Brett R. Levine

**Affiliations:** a Department of Orthopaedic Surgery, University of California, San Francisco Fresno, CA, USA; b Department of Orthopaedic Surgery, Rush University Medical Center, Chicago, IL, USA; c Department of Pathology, Rush University Medical Center, Chicago, IL, USA

**Keywords:** Adverse local tissue reaction (ALTR), Total hip arthroplasty (THA), Ceramic, Complication

## Abstract

Adverse local tissue reaction is an uncommon but frequently described complication after total hip arthroplasty (THA). It is most often associated with metal-on-metal hips and less frequently with metal-on-polyethylene implants as part of a mechanically assisted crevice corrosion process. In this report, we describe a rare case of an atypical adverse local tissue reaction in a patient with a ceramic-on-ceramic THA. Abrasive backside liner wear from a prominent screw head, failure of the liner locking mechanism, and liner fragmentation secondary to component-component impingement created an atypical mass and fluid collection leading to THA failure. This case demonstrates the importance of appropriate cup-liner positioning, thorough workup of pain after THA, and the ability of ceramic debris to cause an associated, atypical adverse local tissue reaction.

## Introduction

Adverse local tissue reaction (ALTR) is a significant complication historically associated with metal-on-metal (MoM) total hip arthroplasty (THA). ALTRs represent a spectrum of reactions due to an inflammatory response to small, often metal, debris created by the articular surface of the implants as well as taper junctions [1,2]. This inflammatory response can be macrophage-induced cytotoxicity stimulated by third-party debris or a type IV delayed hypersensitivity reaction, leading to local damage causing periprosthetic bone and soft-tissue destruction [1,3,4]. These changes can cause a wide array of symptoms and is believed to be the origin of pain, instability, and dysfunction in hip arthroplasty complicated by ALTR [5,6]. More recently, ALTR has become an emerging complication in metal-on-polyethylene implants at the head-neck taper junction secondary to mechanically assisted crevice corrosion [7]. Regardless of the etiology, rare systemic elevation of metal levels has been associated with end-organ deposition or dysfunction, neurogenic toxicity, and cardiac abnormalities [8]. There have been limited reports regarding ALTR in the setting of ceramic THA implants [[9], [10], [11]]. We present a case of an atypical ALTR in a patient with a ceramic-on-ceramic (CoC) THA which was found to have both abrasive backside liner wear from a prominent screw and failed locking mechanism as well as fragmentation of the liner rim due to component-component impingement.

## Case history

**Figure 1:**
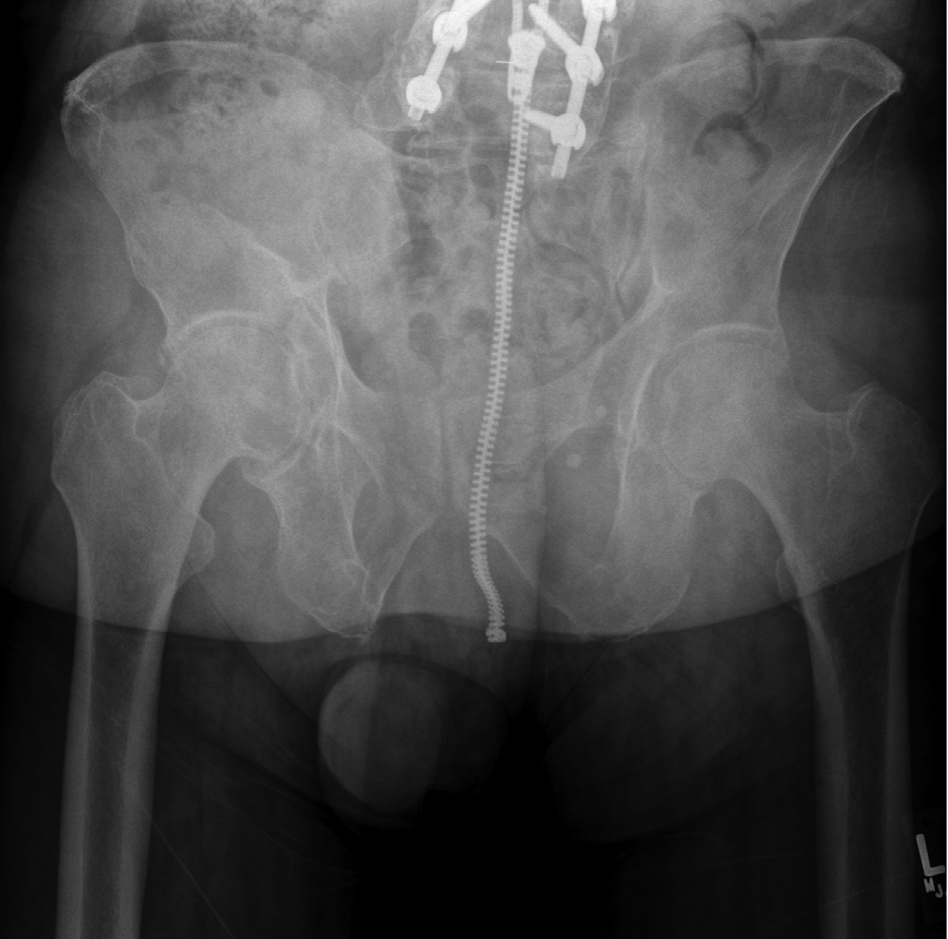
Anteroposterior (AP) pelvis at original presentation showing end-stage right-hip osteoarthritis.

In 2012, a 59-year-old male underwent right THA for end-stage osteoarthritis (Fig. 1). Given the patient’s younger age, the treating surgeon elected to use a cementless THA with a CoC articulation. Using a modified Hardinge approach, the patient received a Lineage 58-mm porous-coated, titanium acetabular component (Wright Medical Group, Memphis, TN) with a 32-mm inner diameter ceramic liner. A Perfecta RS, proximally coated fluted, titanium femoral component (Wright Medical Group, Memphis, TN) was then used, and a +3.5 ceramic head neck assembly was attached. His postoperative course was uneventful without concerns for component complication, malposition, or infection.

**Figure 2:**
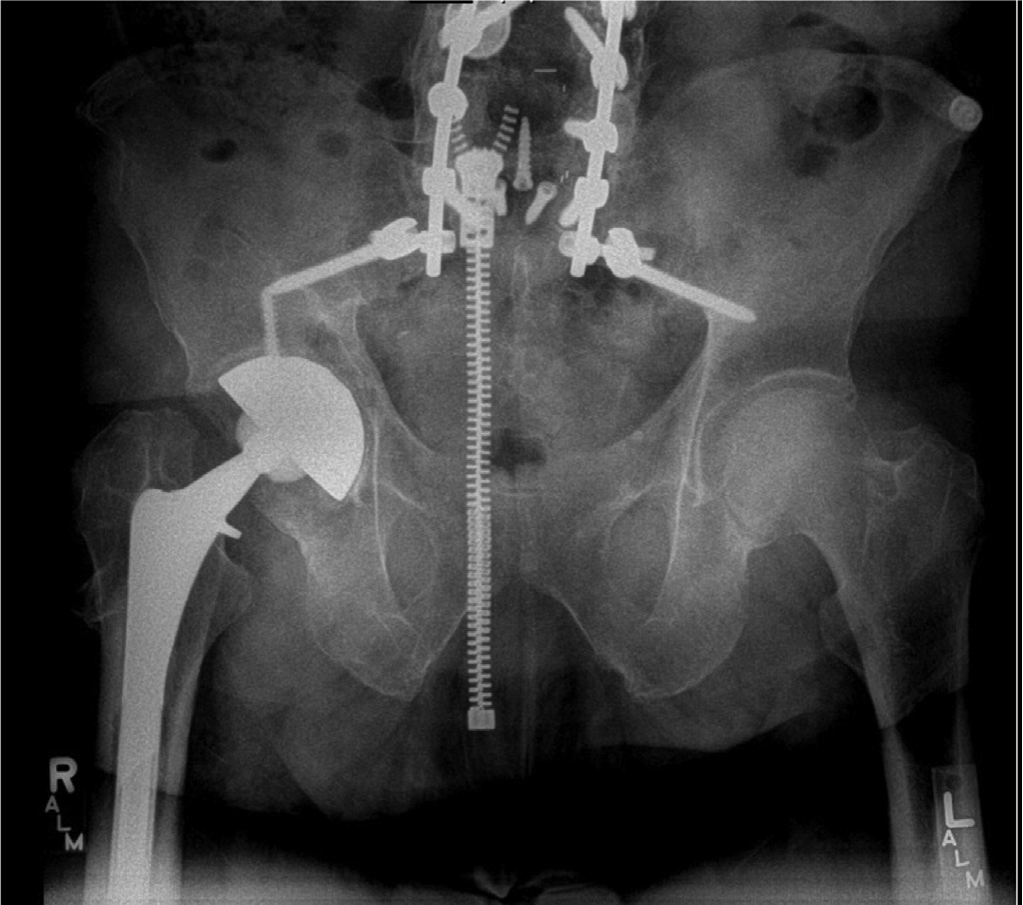
Prerevision AP pelvis illustrating periacetabular radiolucency without any other obvious signs of malalignment or positioning.

**Figure 3:**
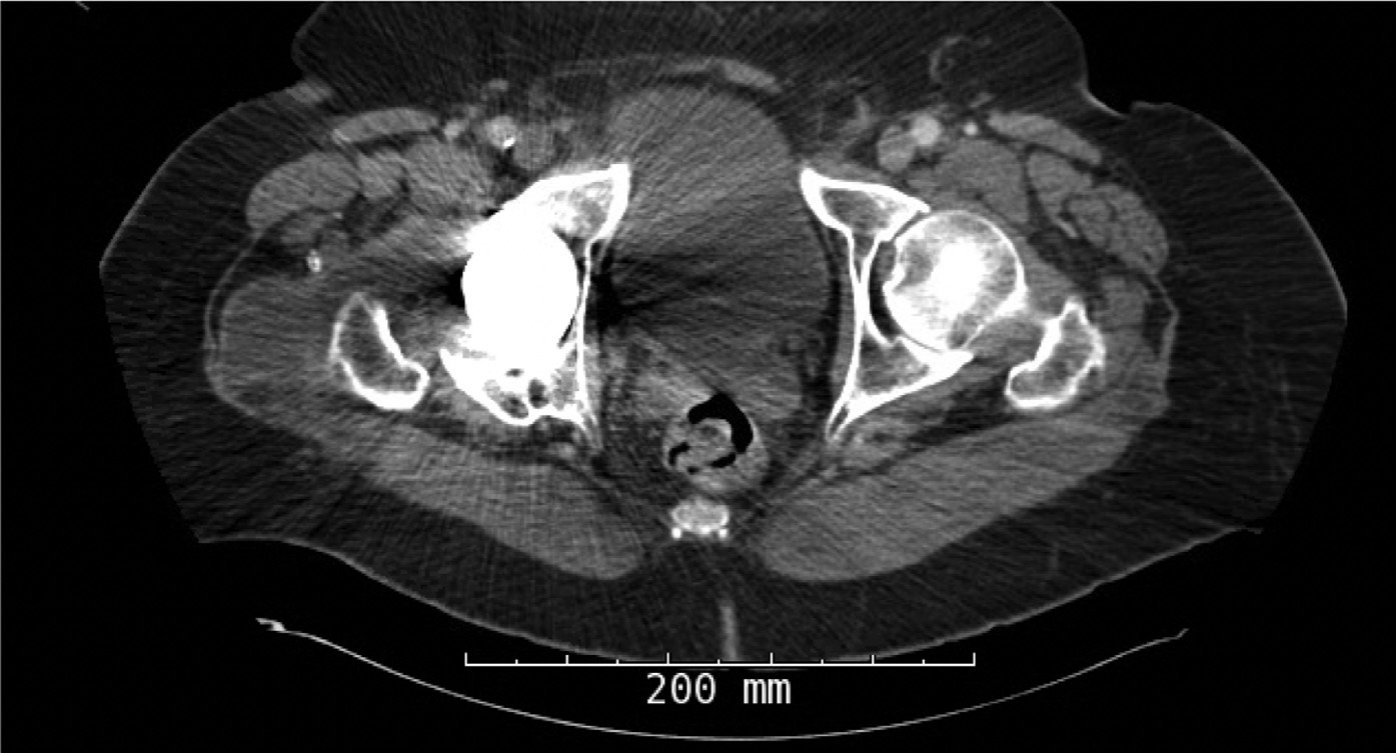
Axial CT scan demonstrating periprosthetic fluid collection.

In September of 2019, now 67 years old, the patient presented back with increasing right hip pain. He reported 3-year history of intermittent dull hip pain localized to the groin causing him to regress and ambulate using a cane. In addition, he complained of progressive squeaking coming from his right hip especially as he transitioned from a seated to a standing position. He further felt like the implant had started shifting inside and developed a reproducible, audible clunk. On physical examination, he had a moderate limp and slightly decreased abductor strength compared with his contralateral side, but a true trendelenburg gait was not appreciated. Review of his radiographs illustrated progressive radiolucency around the acetabular component compared with early postoperative images (Fig. 2). A Technetium 99-m bone scan showed a right hip prosthesis with mild periprosthetic uptake adjacent to the right greater trochanter corresponding to a 4.7 × 6.0-cm fluid collection previously seen on a CT scan ordered by his primary care physician (Fig. 3). Physical examination revealed full extension with flexion of the hip to 95°. He was able to externally rotate to 35° and internally rotate to 10° in a seated position. The previously mentioned clunk was appreciated in the office as he moved his hip from flexion to extension while getting up from a chair.

The initial infectious workup was negative with synovial white blood cell count demonstrating 204 white blood cells, 4000 red blood cells, 33.8% polymorphonuclear cells, and 66.2% mononuclear cells. Erythrocyte sedimentation rate was 20, and C-reactive protein level was <5. Based on his clinical and radiographic parameters, the presumed diagnosis was aseptic loosening of the acetabulum as an ALTR was deemed less likely because of the CoC components. After discussion of the risks and benefits of the procedure as well as research consent, the patient decided to move forward with revision THA.

**Figure 4:**
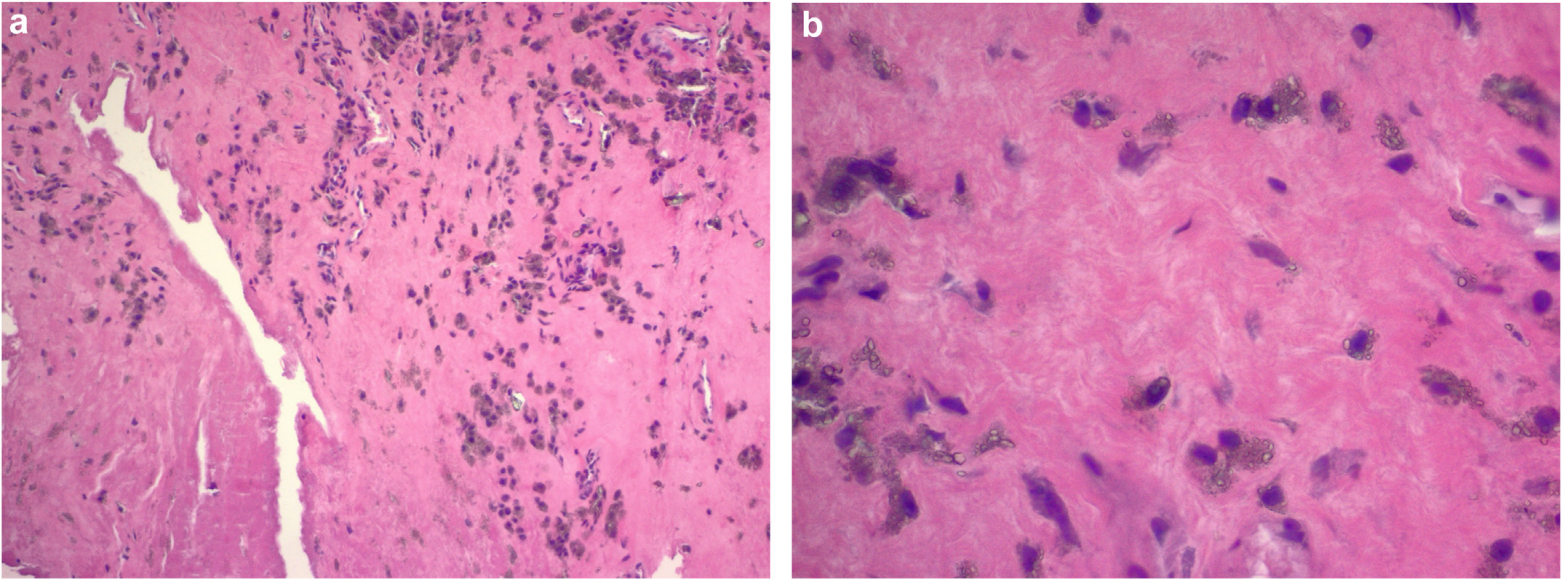
(a) Histology illustrating necrotic synovium lining the crack. Histiocytes containing pigmented and nonpigmented refractile particles are also present in the fibrous stroma. (b) High-magnification slide showing histiocytes containing refractive pigment particles.

**Figure 5:**
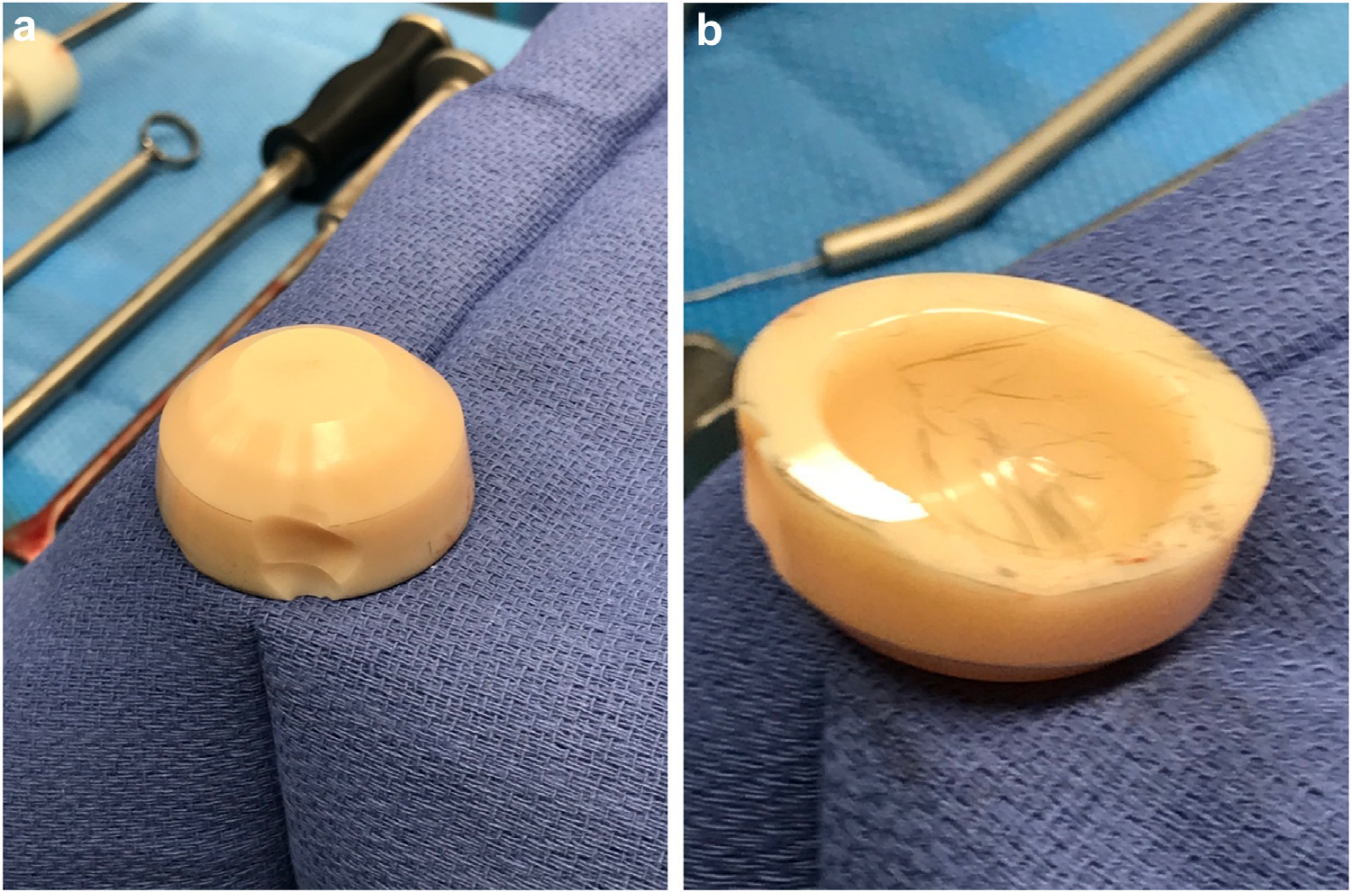
(a & b) Photographs of a chipped ceramic liner with significant backside wear.

During the revision surgery (performed through a priorly modified Hardinge approach), we first obtained fluid from the hip joint, which was sent for cell count with differential and culture, and a portion of the hip capsule was resected and sent for an intraoperative frozen section analysis. The hip was then ranged to assess stability, which illustrated femoral neck-liner contact with extension past the neutral point and any external rotation. The THA was then dislocated to better assess the implant components. The capsule and synovium were noted to be abnormal, and a fluid collection was appreciated as the abductors were elevated from the greater trochanter during the exposure. Visually the tissue looked like a typical ALTR, which was later confirmed by a histological analysis as particle disease (with no evidence of metal debris) with synovial necrosis without any substantial inflammatory reaction (Fig. 4a & b). The ceramic liner was also found to be chipped around the posterior edge, which was the sight of component-component impingement (Fig. 5a). Furthermore, during removal of the liner, it was noted that it had lost its taper fit. This was likely responsible for the significant backside wear (thought to be due to the smooth surface on the liner and absence of a fractured ceramic piece) gouged into the acetabulum indicating that the liner was spinning within the cup in situ (Fig. 5b).

Using a curved osteotome system, we then removed the well-fixed cup and proceeded with reaming of the acetabulum after thorough irrigation and sharp debridement of the hypertrophic synovium and capsule. Once the frozen section and cell count came back negative, we continued with reimplantation of the acetabular component. A 62-mm elliptical, porous tantalum modular cup (Zimmer, Warsaw, IN) was impacted into place in appropriate abduction and anteversion. Adjunct fixation was achieved with five screws in multiple quadrants of the cup. A 40-mm polyethylene liner was placed in the acetabular component and a 40-mm, +7-mm Biolox option (Ceramtec, Plochingen, Germany) femoral head was placed on the femoral trunnion. Of note, the femoral trunnion was inspected, and despite the component-to-component impingement, there was minimal damage to the femur, and we were able to forgo the morbidity of removing this implant. He was ultimately found to have excellent range of motion and stability without impingement intraoperatively.

**Figure 6:**
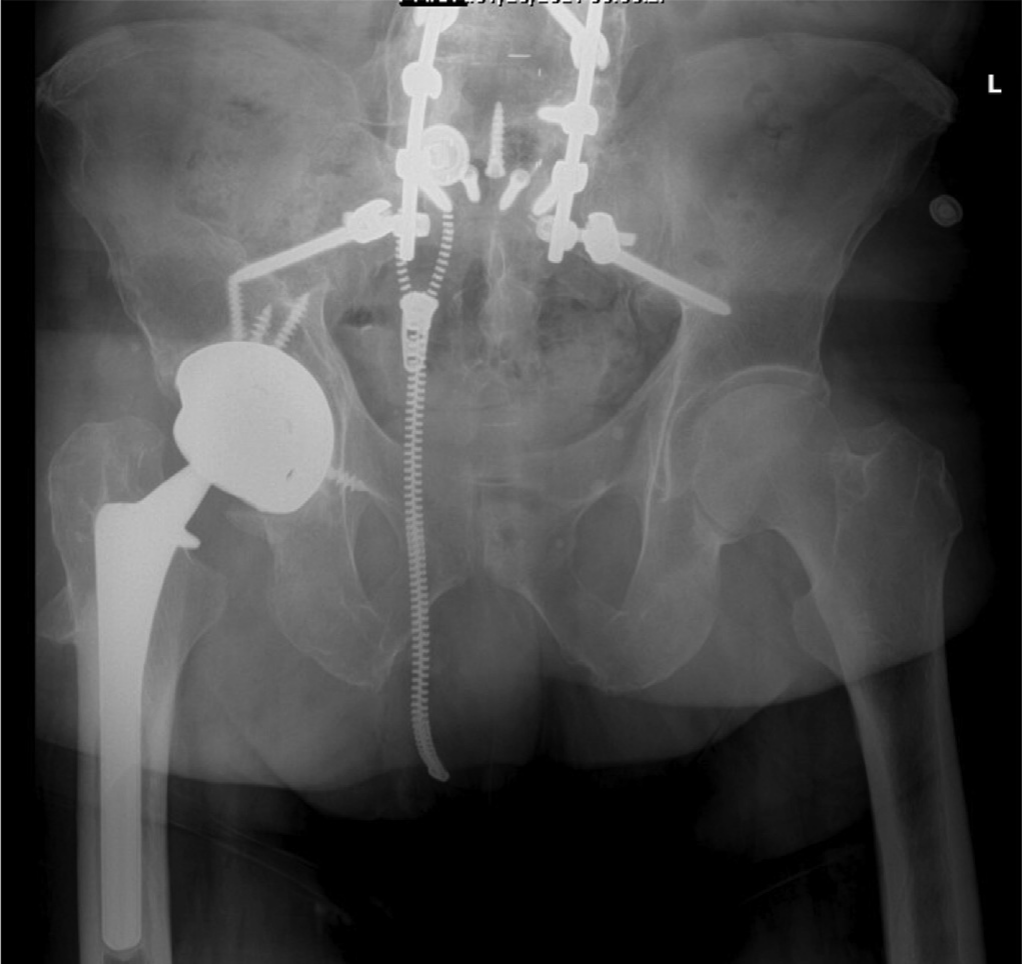
Six-month postoperative AP pelvis illustrating stable components without loosening or other complication.

The patient’s postoperative course was uneventful. He was made 50% weight-bearing with no active abduction immediately after the surgery. He was discharged home on postoperative day 3 and instructed to take oral antibiotics for 2 weeks. Six weeks after the surgery, he was progressed to full weight-bearing, and all precautions were released. At his latest follow-up visit, at 6 months postoperatively, he was doing well without any further complication. Postoperative imaging illustrated well-fixed components without signs of loosening (Fig. 6). He reported 8/10 satisfaction with his surgery, and his UCLA Activity Score was- regularly participates in mild activities. His Harris Hip Score improved from 45 preoperatively to 67 after surgery.

## Discussion

ALTR is a known complication of MoM hip arthroplasty with an incidence of approximately 5% and mechanically assisted crevice corrosion specifically approaching 2% [2,[12], [13], [14]]. ALTRs are described as the growth of cystic or solid fibrotic masses originating in the synovial membrane of patients with hip implants [15]. In patients with excessive articular wear, the etiology is believed to stem from the generation of metal ions, mainly cobalt, inducing mitochondrial stress generating a hypoxic-like condition in cells. This subsequently triggers the synthesis and secretion of cytokines that elicit inflammation in periprosthetic tissues [[16], [17], [18]]. An alternative but possibly interrelated mechanism is associated with a lymphocyte-driven hypersensitivity reaction more commonly seen in low-wear implants. In our unique case, the former pathway can likely be implicated in the formation of the atypical ALTR.

Fracture or fragmentation of the ceramic bearing surfaces is an important complication associated with CoC articulations. Fortunately, owing to improvements in materials, designs, manufacturing processes, and surgical technique, fractures have become relatively uncommon with an estimated occurrence in 0.013% to 1.1% of cases [[19], [20], [21], [22]]. While, ceramic head fractures are catastrophic events, ceramic liner fracture, as seen in our case, can present more discreetly. Component fractures can be caused by trauma, interposition of debris between the neck taper or acetabular cup and the ceramic component, or improper handling during implantation [23]. However, ceramic liners are at highest risk of fracture when malpositioned or malseated in the acetabular component [24]. In our case, there was fragmentation at the liner secondary to impingement at the femoral neck on the liner edge during hip extension-external rotation as well as a smooth gouge out of the ceramic liner related to back side wear. There was also a mildly prominent screw head that correlated to this area of material loss on the liner. This was an interesting finding as the screw head illustrated minimal material loss, while the brittle ceramic had signs of wear without any metallic tissue staining that is often seen with titanium component debris. It is likely that this or the presumed malseating of the liner caused secondary incongruence between the liner and the cup possibly leading to a failed locking mechanism and the eventual loosening of the liner. However, if malseating of the liner was present, component liner fracture would be expected and would not appear to be well seated at the time of the revision surgery. In addition, abrasive third-body wear between the liner and femoral head occurred due to component-component impingement and fragmentation of the liner, as illustrated in Figure 4.

Although ceramic is touted for its mostly benign features, its wear particles are not completely unreactive. Mochida et al. found that while CoC THA produces particles at a significantly lower rate than ceramic-on-polyethylene hips, the ceramic debris still elicits a predominately macrophage response, which was confirmed with immunohistochemical staining [25]. Petit et al. illustrated in vitro that macrophage injection of ceramic particles increases proinflammatory cytokine production [26]. Furthermore, macrophage apoptosis was sooner and more significant after ceramic particle ingestion than polyethylene debris [26].

There is a broad range of histologic patterns associated with ALTR, with most lesions classified into four groups: macrophage dominated, mixed macrophage lymphocytic with or without hypersensitivity features, and granulomatous pattern [27]. The histology in this case revealed macrophage-dominant particle diseases with synovial necrosis most consistently seen in MoM bearing surfaces. In this instance, we hypothesize that surface wear between the liner and acetabular cup and prominent screw head generated nanoparticle-size wear debris causing a phagocytotic response. This process leads to macrophage apoptosis as well as exfoliation of necrotic cellular debris and phagocytized secondary wear particles into the joint fluid and manifests clinically years after implantation as seen in our case.

ALTR associated with ceramic components has been previously described in the literature. Most of these reports involved ceramic-on-polyethylene articulations with elevated metal ion levels attributed to the neck or stem [11,27]. There is only one other reported case of pseudotumor formation associated with CoC articulation; however, the case presented here is the first report of an atypical ALTR due to a combination of liner fragmentation and a prominent screw causing ceramic liner locking mechanism failure and backside wear [9].

Thus, ALTR etiologies can be due to a myriad of different causes such as component malposition, articular wear, corrosion, and patient hypersensitivity. While rare, ALTR should remain in the differential diagnosis and workup in symptomatic patients with a CoC hip articulation. With the increasing popularity of ceramic femoral heads, further studies are warranted into the investigation of the natural inflammatory response to ceramic debris from THA implants.

## Summary

ALTR is an uncommon complication after THA, most often associated with MoM hips. Here, a rare case of an atypical ALTR in a patient with a CoC THA is described. Abrasive backside liner wear from a prominent screw head, failure of the liner locking mechanism, and liner fragmentation secondary to component-component impingement created an atypical mass and fluid collection leading to THA failure. This case demonstrates the importance of appropriate cup-liner positioning, thorough workup of pain after THA, and the ability of ceramic debris to cause an associated ALTR.

## Conflicts of interest

B. R. Levine is a paid consultant for Link, Exactech, and Merete; receives royalties or financial or material support from Human Kinetics, Wolers Kluwer, and Elsevier; is in the publications or editorial board of JOA, Orthopedics, and *Arthroplasty Today*; and is a part of the AAHKS Patient education committee, MAOA Education Committee, and AAOS ALI3. All other authors have no conflicts to disclose.

For full disclosure statements refer to https://doi.org/10.1016/j.artd.2022.01.025.

## Informed patient consent

The authors confirm that written informed consent has been obtained from the involved patient, and he has given approval for this information to be published in this case report.

Please refer to Elsevier's policy regarding written patient consent requirements

https://www.elsevier.com/about/policies/patient-consent#:∼:text = That%20individual%2C%20legal%20guardian%20or,writing%20of%20all%20such%20conditions

## Appendix A Supplementary data

Conflict of Interest Statement for Levine

Conflict of Interest Statement for Miller

Conflict of Interest Statement for Patel

Conflict of Interest Statement for Movassaghi
