# Supplementary material for: An Atypical Adverse Local Tissue Reaction After Ceramic-on-Ceramic Primary Total Hip Arthroplasty
Source: Arthroplast Today. 2022 Feb 28;14:71–5. doi: 10.1016/j.artd.2022.01.025 (PMC8889265; doi:10.1016/j.artd.2022.01.025)
Supplement: Conflict of Interest Statement for Levine [file mmc1.pdf]

CONFLICT OF INTEREST STATEMENT

American Association of Hip and Knee Surgeons

(Adopted from the American Academy of Orthopaedic Surgeons disclosure statement)

The following form **must be filled out completely and submitted by each author (example, 6 authors, 6 forms). All items require a response. If there is no relevant disclosure for a given item, enter "*None*."**

Manuscript Title

1. Royalties from a company or supplier (The following conflicts were disclosed)
none

2. Speakers bureau/paid presentations for a company or supplier (The following conflicts were disclosed)
none

3A. Paid employee for a company or supplier (The following conflicts were disclosed)
none

3B. Paid consultant for a company or supplier (The following conflicts were disclosed)
Link, Exactech, Merete

3C. Unpaid consultants for a company or supplier (The following conflicts were disclosed)
none

4. Stock or stock options in a company or supplier (The following conflicts were disclosed)
none

5. Research support from a company or supplier as a Principal Investigator (The following conflicts were disclosed)
none

6. Other financial or material support from a company or supplier (The following conflicts were disclosed)
none

7. Royalties, financial or material support from publishers (The following conflicts were disclosed)
Human Kinetics, Wolters Kluwer, Elsevier

8. Medical/Orthopaedic publications editorial/governing board (The following conflicts were disclosed)
JOA, Orthopedics, Arthroplasty Today

9. Board member/committee appointments for a society (The following conflicts were disclosed)
AAHKS Patient education committee, MAOA Education Committee, AAOS ALI3

Each author must sign AND print or type his/her name, date and submit a separate form

In addition, one BLINDED Conflict of Interest form (no author names used) should be submitted per manuscript with all author disclosures.

Brett Levine		9/21/2021
Author Name (Print or Type)	Author Signature	Date
